# Using automated imaging to interrogate gonadotrophin-releasing hormone receptor trafficking and function

**DOI:** 10.1016/j.mce.2010.07.008

**Published:** 2011-01-15

**Authors:** S.P. Armstrong, C.J. Caunt, A.R. Finch, C.A. McArdle

**Affiliations:** a University of Bristol, School of Clinical Sciences, Labs. for Integrative Neuroscience and Endocrinology, 1 Whitson Street, Bristol BS1 3NY, UK; b Department of Biology & Biochemistry, University of Bath, Claverton Down, Bath BA2 7AY, UK

**Keywords:** Gonadotrophin-releasing hormone, Desensitisation, Trafficking, Imaging, Pulsatility

## Abstract

Gonadotrophin-releasing hormone (GnRH) acts via seven transmembrane receptors on gonadotrophs to stimulate gonadotrophin synthesis and secretion, and thereby mediates central control of reproduction. Type I mammalian GnRHR are unique, in that they lack C-terminal tails. This is thought to underlie their resistance to rapid homologous desensitisation as well as their slow rate of internalisation and inability to provoke G-protein-independent (arrestin-mediated) signalling. More recently it has been discovered that the vast majority of human GnRHR are actually intracellular, in spite of the fact that they are activated at the cell surface by a membrane impermeant peptide hormone. This apparently reflects inefficient exit from the endoplasmic reticulum and again, the absence of the C-tail likely contributes to their intracellular localisation. This review is intended to cover some of these novel aspects of GnRHR biology, focusing on ways that we have used automated fluorescence microscopy (high content imaging) to explore GnRHR localisation and trafficking as well as spatial and temporal aspects of GnRH signalling via the Ca^2+^/calmodulin/calcineurin/NFAT and Raf/MEK/ERK pathways.

## 1 Introduction

GnRH (pGlu-His-Trp-Ser-Tyr-Gly-Leu-Arg-Pro-Gly-NH_2_, also known as GnRH I) stimulates secretion of luteinising hormone (LH) and follicle-stimulating hormone (FSH) from gonadotrophs, and thereby mediates central control of reproduction. It acts primarily via Gαq-coupled seven transmembrane (7TM) receptors to stimulate phospholipase C, with consequent mobilisation of Ca^2+^, which mediates acute stimulation of exocytotic gonadotrophin secretion. It also activates protein kinase C (PKC) isozymes and mitogen-activated protein kinase (MAPK) cascades which (together with Ca^2+^/calmodulin and its effectors), control gonadotrophin synthesis (Stojilkovic and Catt, 1995b; Millar et al., 2004; Cheng and Leung, 2005). Most vertebrates also express the highly conserved GnRH II ([His^5^, Trp^7^, Tyr^8^]GnRH I) and ligand selective receptors have evolved in parallel with these distinct forms of GnRH. Mammalian type I GnRHR are selective for GnRH I and lack C-terminal tails (Millar et al., 2004; Cheng and Leung, 2005). This unique structural feature has major implications for receptor function and also offers some informative research strategies. In recent years we have increasingly used imaging readouts for interrogation of GnRH function. The array of fluorescent labelling reagents now available to researchers allows quantification of events in intact fixed and live cells in imaging assays, which can readily be expanded to integrate complex information by using multiple fluorophore reporters (Pepperkok and Ellenberg, 2006; Lang et al., 2006). The recent development of affordable high content microscopy (HCM) platforms permits automated image acquisition from cells and tissues in multiwell plate formats allowing efficient capture of single cell information, such as subcellular protein compartmentalisation (Pepperkok and Ellenberg, 2006; Lang et al., 2006). Stored images can then be analysed using pre-defined software algorithms, allowing the whole workflow to become unbiased and automated. The development of this technology has largely been driven by the need for improved secondary screening in drug discovery, but the increased throughput and statistical power of these approaches has seen their increasing adoption by academic laboratories (Pepperkok and Ellenberg, 2006; Lang et al., 2006). This dovetails with the rapid increase in free bioinformatic data and the affordability of large-scale plasmid, small molecule and RNA interference libraries. In this review we will outline some of the HCM approaches we have developed to interrogate GnRHR trafficking and signalling.

## 2 Desensitisation and internalisation

For many 7TM receptors, agonist-activated receptor conformations are substrates for G-protein receptor kinases. This phosphorylation occurs most often within the receptor's carboxy-terminal tail (C-tail), and facilitates binding to β-arrestins that mediate receptor desensitisation and internalisation, as well as signalling to arrestin-scaffolded effectors (Pierce and Lefkowitz, 2001; Luttrell and Lefkowitz, 2002). Agonist-induced phosphorylation, arrestin binding, arrestin-mediated desensitisation, internalisation and signalling have all been shown with non-mammalian GnRHR (e.g. catfish or Xenopus GnRHR, both of which have C-tails with multiple potential phosphorylation sites) but not for tailless type I GnRHR (Heding et al., 2000; McArdle et al., 2002; Ronacher et al., 2004; Hislop et al., 2005; Caunt et al., 2006b). Thus the advent of type I mammalian GnRHR has been associated with the loss of functionally relevant C-tails (Davidson et al., 1994; McArdle et al., 1995, 1996; Heding et al., 1998; Blomenrohr et al., 1999; Willars et al., 1999; Hislop et al., 2000; Vrecl et al., 2000; Heding et al., 2000; Willars et al., 2001; McArdle et al., 2002; Millar et al., 2004). Lack of type I mammalian GnRHR desensitisation is intriguing in light of the fact that sustained stimulation causes desensitisation of GnRH-stimulated gonadotrophin secretion. Moreover, this desensitisation underlies the therapeutic use of GnRH agonists in clinical treatment (Conn et al., 1987; Stojilkovic and Catt, 1995a; McArdle et al., 2002; Millar et al., 2004; Cheng and Leung, 2005). Desensitisation of GnRH-stimulated gonadotrophin secretion must be due to down-stream adaptive changes, making type I GnRHR an excellent model for exploration of such changes in the absence of direct receptor desensitisation. For example, we have previously shown that sustained GnRH treatment caused a pronounced down-regulation of inositol 1,4,5 trisphosphate (IP_3_) receptors and consequent desensitisation of GnRH effects on the cytoplasmic Ca^2+^ concentration [Ca^2+^]_i_ (Willars et al., 2001; McArdle et al., 2002). Other mechanisms that may contribute to desensitisation of GnRH effects on [Ca^2+^]_i_ and/or exocytotic gonadotrophin secretion include GnRH-mediated desensitisation of voltage-operated Ca^2+^ channels (Stojilkovic and Catt, 1995a) and agonist-induced GnRHR internalisation (below). In the long-term, depriving gonadotrophs of the pulsatile GnRH needed for efficient transcription of the genes encoding the GnRHR and gonadotropin subunits (below) may contribute to or explain, the chemical castration caused by GnRH agonists *in vivo* (Huhtaniemi et al., 2009).

## 3 GnRHR as intracellular proteins

One of the most surprising recent discoveries in this field is that human (h)GnRHR are largely intracellular. This developed from work on GnRHR point mutations that cause infertility (hypogonadotropic hypogonadism). Although initially thought to perturb signalling, it was found that most of these mutations actually impair trafficking and reduce the cell surface number of GnRHR (Brothers et al., 2004). Moreover, a membrane permeant non-peptide GnRHR antagonist (IN3) could facilitate signalling via most of these mutant hGnRHR (Janovick et al., 2002, 2003; Ulloa-Aguirre et al., 2004; Brothers et al., 2006; Conn et al., 2006; Conn et al., 2007; Conn and Janovick, 2009). By analogy with other 7TM receptors (Petaja-Repo et al., 2000; Edwards et al., 2000; Petaja-Repo et al., 2001; Tan et al., 2004; Bernier et al., 2004; Dong et al., 2007), this antagonist is thought to act as a pharmacological chaperone, enabling the conformational change in GnRHR required for trafficking from the endoplasmic reticulum (ER) to the surface. Consistent with this, it was found that calnexin, a major component of the cell's ER exit quality control system, was able to bind to GnRHR and reduce functional GnRHR expression at the cell surface (Brothers et al., 2006; Yanez and Conn, 2010). Interestingly, the non-peptide antagonist also caused a modest increase in signalling via wild-type hGnRHR suggesting that there is a significant reserve of potentially functional wild-type hGnRHR within the cell.

**Fig. 1:**
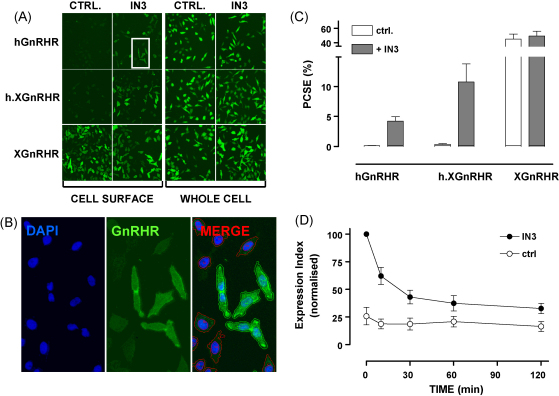
An automated imaging assay for GnRHR quantification. Cells grown in 96 wells were transduced with Ad expressing N-terminal HA-tagged hGnRHR, XGnRHR or h.XGnRHR then incubated ∼20 h with 0 or 1 μM of the non-peptide antagonist IN3 before indirect fluorescence labelling of cell surface receptors (primary antibody added to intact cells) or whole cell receptors (primary antibody added after permeabilisation). Nuclei were also stained with DAPI and digital images were captured using a 10× objective and a 0.6 mm^2^ field of view. Panel A shows representative images (each approximately 25% of the field captured) of whole cells and cell surface staining in cells transduced with the indicated receptors. Panel B shows a higher power image of nuclei, HA-XGnRHR and merged stains from the boxed region in panel A. It also illustrates the automated image segmentation used to define perimeters of nuclei (blue) and cells (green or red) and application of a filter to distinguish cells in which staining was >10% above background (green perimeters) or <10% above background (red perimeters). Receptors can be quantified by calculation of an expression index (EI = % +ve stained cells × mean fluorescence intensity in those cells) and proportional cell surface expression (PCSE) is calculated as the cell surface EI as a % of the whole cell EI. Panel C shows PCSE values calculated from the same representative experiment as used for panels A and B. Panel D shows the cell surface EI for HA-hGnRHR in control and IN3 pre-treated cells, stimulated for the indicated period with 10^−7^ M GnRH. Note that the agonist-induced reduction in cell surface hGnRHR expression is only evident in IN3 pre-treated cells.

Adapted from Finch et al. (2009).

The ability of pharmacological chaperones to increase cell surface hGnRHR expression was initially inferred from increased GnRH-stimulated [^3^H]IP_*x*_ accumulation (Janovick et al., 2002, 2003; Ulloa-Aguirre et al., 2004; Brothers et al., 2006; Conn et al., 2006, 2007) and then documented by microscopy. The latter approach has been hampered by the lack of validated antibodies to normal GnRHR, so we have developed models based on adenovirus (Ad)-mediated expression of GnRHR with N-terminal (exofacial) haemagglutinin (HA) epitope tags and indirect immuno-fluorescence staining coupled with automated image acquisition and analysis. Using this we simply quantified HA-GnRHR staining at the cell surface (anti-HA added to intact cells) and the whole cell (anti-HA added to permeabilised cells) for a range of GnRHR constructs and cell types incubated with or without IN3 (Finch et al., 2008, 2010). We calculated whole cell and cell surface expression indices (% positive cells x mean fluorescence intensity in those cells) and used these to determine the proportional cell surface expression (PCSE) as shown in Fig. 1. This revealed that a remarkably small proportion of HA-hGnRHR is located at the cell surface (PCSE <1% in most cell types tested). In contrast, the PCSE of a non-mammalian GnRHR (the XGnRHR) was much higher (40–60%) and addition of the XGnRHR C-tail to the hGnRHR (h.XGnRHR) increased PCSE approximately 5-fold. Accordingly, the absence of any C-terminal tail may also contribute to this unusual aspect of hGnRHR function, although other structural features including a primate-specific Lys191 (Ulloa-Aguirre et al., 2004; Conn et al., 2007) and a second extracellular glycosylation site (Davidson et al., 1995) are undoubtedly also involved.

The low proportion of HA-hGnRHR at the cell surface (<1% in MCF7 cells) is remarkable in light of the robust hGnRHR-mediated [^3^H]IP_*x*_ accumulation seen in these cells (Finch et al., 2004, 2008). This led us to suspect that the HA-tag was influencing receptor function (Brothers et al., 2003) but we have found no effect of the tag on binding affinity or specificity in binding assays or on ligand potency and specificity in functional assays (Finch et al., 2008, 2010). We were also concerned that the imaging assay simply quantifies the proportion of HA tag at the cell surface and that this might not equate to functional GnRHR but we have found effects of IN3 on receptor expression are paralleled by effects on receptor function in a number of assays. These include GnRHR-mediated [^3^H]IP accumulation assays (Finch et al., 2008) and GnRHR-mediated NFAT2-EFP (nuclear factor of activated T cells-emerald fluorescent protein) translocation assays (Finch et al., 2009) as well as antiproliferative and pro-apoptotic effects of GnRHR activation in MCF7 cells (Finch et al., 2008). In each case, IN3 behaves as a competitive GnRHR antagonist, but when long-term co-incubation protocols are used the IN3 increases cell surface hGnRHR expression and can actually increase GnRH effects. These functional data parallel the imaging, supporting the notion that the majority of hGnRHR are located in a potentially functional intracellular pool that can be brought to the cell surface by pharmacological chaperones. Together, the co-localisation studies and effects of pharmacological or biochemical chaperones suggest that these intracellular hGnRHR are primarily within the ER (Brothers et al., 2004, 2006; Sedgley et al., 2006) although there are presumably also hGnRHR within retrograde and/or anterograde transport vesicles and early studies suggested their presence in the nucleus or nuclear envelope (Millar et al., 1983; Halmos and Schally, 2002) as recently demonstrated for epitope-tagged GnRHR (Re et al., 2010).

## 4 Agonist-induced GnRHR down-regulation and trafficking

**Fig. 2:**
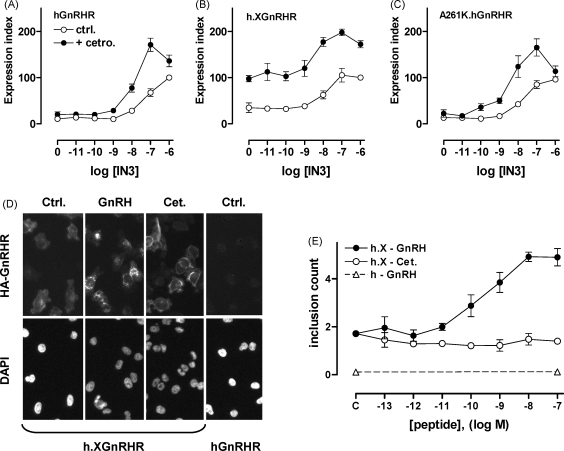
Peptide and non-peptide antagonist effects on GnRHR localisation. Panels A–C: HeLa cells transduced with Ad HA-hGnRHR, h.XGnRHR or A261K-hGnRHR were incubated ∼20 h in medium with the indicated concentration of IN3 with 0 (ctrl.) or 10^−7^ M cetrorelix (cet.) before determining the cell surface expression index, as above. Note that cetrorelix had no effect on HA-hGnRHR alone, but synergised with IN3 to increase cell surface expression of hGnRHR. Similar effects were seen in cells expressing the signalling-deficient A261K hGnRHR mutant, demonstrating that the IN3 and cetrorelix effects on cell surface hGnRHR expression are not dependent upon G-protein activation. Panel D: cells transduced with Ad HA-h.XGnRHR or hGnRHR were incubated for 60 min with anti-HA at 21 °C. They were then washed and incubated for 60 min with 10^−7^ M GnRH or cetrorelix, or without test compound (ctrl.) before fixation and staining (DAPI and anti-HA). In HA-h.XGnRHR expressing cells GnRH caused an increase in bright punctate anti-HA staining (indicating agonist-induced receptor internalisation into endosomes) and this was measured using a granularity assay to quantify “inclusions” (panel E). Note that inclusions were not measurable in HA-hGnRHR expressing cells because there are too few hGnRHR at the cell surface for efficient labelling during the low temperature loading period. However, GnRH caused a dose-dependent increase in inclusion count in HA-h.XGnRHR expressing cells, whereas cetrorelix had the opposite effect. The implication is that the peptide increases cell surface hGnRHR expression by slowing its internalisation from the cell surface.

Adapted from Finch et al. (2009).

The discovery that hGnRHR are largely intracellular also has important implications for understanding trafficking from the cell surface. It is often assumed that agonist-induced internalisation and down-regulation contributes to the efficacy of GnRH agonists in cancer therapy but there is very little direct evidence for such regulation of hGnRHR. Extrapolation from the early studies performed primarily with rodent GnRHR (Schvartz and Hazum, 1987; Lin et al., 1998; Petaja-Repo et al., 2000; Pierce et al., 2002) is less compelling in light of the known differences between rodent and hGnRHR compartmentalization (and hence trafficking (McArdle et al., 1995, 2002)), and a recent study (monitoring uptake of radiolabelled antibodies targeting tagged GnRHRs) revealed that type I mammalian GnRHR undergo constitutive but not agonist-induced internalisation in COS-7 or HEK293 cells (Pawson et al., 2008). With this in mind we adapted our automated imaging methods to monitor cell surface expression and trafficking of HA-tagged GnRHR. We found that GnRH II rapidly reduces cell surface XGnRHR expression, and that GnRH reduces cell surface mouse (m)GnRHR and h.XGnRHR but saw no effect of GnRH on cell surface hGnRHR, which is not surprising given the low cell surface expression of this receptor in unstimulated cells (Fig. 1). However, when cells were pre-treated with IN3 to increase cell surface receptor expression, subsequent GnRH addition did cause a pronounced reduction in cell surface hGnRHR, h.XGnRHR and mGnRHR (Fig. 1 and (Finch et al., 2009, 2010)). This down-regulation of cell surface hGnRHR was dependent upon signalling because no such effect was seen in cells expressing a mutant of the hGnRHR (A261K) that does not activate its cognate G-protein (Myburgh et al., 1998), and was also reversed by addition of cetrorelix 2 h after the agonist (Finch et al., 2009, 2010). We also used antibody loading and automated imaging to monitor receptor trafficking to punctate regions within the cells (presumably endosomes). These “granularity assays” revealed that agonists stimulate the trafficking of hGnRHR, h.XGnRHR, mGnRHR and XGnRHR (but not A261K-h.XGnRHR) and the internalisation of h.XGnRHR. Using fluorescent transferrin to label endosomes, we found that agonists stimulate redistribution of hGnRHR and h.XGnRHR to punctuate regions where they are co-localised with transferrin. Similar data have been previously seen by confocal microscopy (i.e. for HA-tagged rat GnRHR in HEK293 cells, (Vrecl et al., 2000)) supporting the notion that GnRH also stimulates the redistribution of HA-GnRHR from the cell surface to endosomes in the HeLa cell model. However, our internalisation assay was dependent upon loading of anti-HA to cell surface HA-GnRHR at low temperature and labelling was too low for imaging of the hGnRHR (because cell surface receptor expression was low). Consequently, we could demonstrate agonist-induced hGnRHR trafficking but could not test for agonist-induced hGnRHR internalisation (Fig. 2). Nevertheless, the parallel effects of agonist on cell surface hGnRHR, mGnRHR and h.XGnRHR, as well as their trafficking and internalisation (where measurable) clearly support the notion that agonists can reduce cell surface hGnRHR number by stimulating hGnRHR internalisation.

## 5 Ligand biased efficacy

Conventional receptor theory assumes that there are single inactive and active receptor conformations, and that antagonists occupy the former, whereas agonists induce or stabilise the latter. It is increasingly recognised, however, that there are multiple active conformations for many (probably all) 7TM receptors (Galandrin and Bouvier, 2006; Kenakin, 2007). The overriding reason for interest in multiple active 7TM receptor conformations is that these distinct conformations may not only be preferentially induced or stabilised by different ligands but may also couple differentially to distinct effectors. This provides the basis for “ligand-biased efficacy” (also known as “ligand-directed trafficking of receptor signalling”) that has recently been reported for GnRHR. Thus we found that PKC activation increased affinity of XGnRHR for GnRH II but not for buserelin, demonstrating the existence of multiple active GnRHR conformations (Caunt et al., 2004), and different active conformations of rat and hGnRHR are thought to mediate antiproliferative effects and G_q/11_ activation in some models (Lopez de et al., 2008). The effect of non-peptide antagonists on cell surface expression of GnRHR demonstrates the existence of multiple GnRHR conformations (that do or do not traffic efficiently to the cell surface) within the cell. Although it is not clear how these are related to active conformations at the cell surface, recent work comparing effects of peptide and non-peptide antagonists on GnRHR expression is more directly pertinent to this issue. Using the imaging assays above, we found that IN3 increased the number and proportion of HA-hGnRHR at the cell surface in MCF7 (breast cancer) cells, and that this effect was not mimicked or blocked by peptide antagonists (antide and cetrorelix). This is entirely consistent with IN3 acting intracellularly to facilitate hGnRHR trafficking to the cell surface, and the membrane impermeant peptide antagonists being ineffective because they do not access the intracellular site of IN3 action. However, we were surprised to find that the peptide antagonists did cause a modest increase in cell surface expression of the h.XGnRHR (Finch et al., 2008). We reasoned that the peptides might do so by slowing internalisation from the cell surface and that their effect would therefore only be evident when there are appreciable numbers of receptors at the surface. To address this we performed similar experiments in HeLa cells (PCSE values for hGnRHR and h.XGnRHR are higher in HeLa than in MCF7 cells) and also tested for possible interaction between peptide and non-peptide antagonists. We found that antide and cetrorelix have comparable efficacy and greater potency than IN3 at increasing cell surface h.XGnRHR expression in HeLa cells (Finch et al., 2010). Although these peptides had no measurable effect on cell surface hGnRHR expression alone, they did synergize with IN3 to increase cell surface hGnRHR expression and did increase mGnRHR expression ((Finch et al., 2010) and Fig. 2). They also slowed h.XGnRHR internalisation, as measured using the granularity assay described above ((Finch et al., 2010) and Fig. 2) and increased cell surface expression of hGnRH and mGnRHR in LβT2 gonadotroph cells. Thus it appears that the two types of antagonist have the potential to increase cell surface GnRHR number in different ways; the membrane permeant non-peptide antagonist acting within the cell to accelerate trafficking to the PM, and the membrane impermeant peptide acting at the cell surface to slow trafficking from the PM (Table 1). These data demonstrate an unexpected feature of the peptide antagonists. In functional assays reporting G_q/11_ activation ([^3^H]IP_*x*_ accumulation and NFAT-EFP translocation), GnRH and buserelin are full agonists and cetrorelix is thought to be a full antagonist (33,34), influencing receptor function solely by inhibiting agonist effects. In contrast, in the HA-h.XGnRHR internalisation assay, GnRH and buserelin are agonists but cetrorelix acts as an inverse agonist, reducing internalisation in the absence of GnRH. Most importantly, this data reveals the existence of an antagonist-occupied GnRHR conformation at the cell surface that differs from that of the unoccupied receptor, and demonstrates the occurrence of ligand-biased efficacy at GnRHR with therapeutically relevant ligands (cetrorelix and buserelin), normal (i.e. non-tailed) receptors and in gonadotroph lineage cells (Table 1). Such effects may also be pertinent to GnRHR signalling in non-pituitary sites (i.e. in hormone-dependent cancers) where coupling to effectors other than G_q/11_ may occur, and effects of agonists have been found to be mimicked rather than blocked by peptide antagonists (Eidne et al., 1987; Imai et al., 1997; Emons et al., 1998; Limonta et al., 2003; Moretti et al., 2003; Maudsley et al., 2004).

## 6 GnRHR signalling TO ERK

Like many other 7TM receptors, GnRHR activate the extracellular signal-regulated kinase (ERK) cascade (Caunt et al., 2006a). In many cell models of GnRH signalling, ERK controls the transcription of both LH and FSH, which in turn regulate fertility. This appears to reflect the *in vivo* scenario, as pituitary-specific removal of ERK1/2 in mice reduces LH synthesis and causes female infertility (Bliss et al., 2009). Cellular context can also have a large influence on mechanisms of ERK activation by GnRH. In gonadotroph-lineage cells, ERK activation typically occurs through PKC-dependent activation of Raf (Liu et al., 2002), but in some cells of neuronal origin, PKC-dependent transactivation of epidermal growth factor (EGF) receptors provides the main route of ERK activation (Shah et al., 2003a,b). As noted above, one of the peculiar features of type I mammalian GnRHR is that they lack the C-terminal intracellular tails required to mediate β-arrestin binding and receptor desensitisation (Heding et al., 2000; McArdle et al., 2002; Ronacher et al., 2004; Hislop et al., 2005; Caunt et al., 2006b). Non-mammalian GnRHR can bind β-arrestin and can signal to ERK in the cytoplasm via arrestin-dependent routes, while mammalian type I GnRHR appear to utilise other scaffolds to control the kinetics and compartmentalisation of ERK signals (Caunt et al., 2006a,b). Recent studies in gonadotrophs have described cytoplasmic signalling scaffolds that are necessary for GnRH signalling to ERK in caveolin rich lipid rafts (Navratil et al., 2003). Studies have additionally shown that paxillin and Pyk2 can act as scaffolds within focal adhesions, which serve to form a complex of ERK activating proteins and regulators (such as PKC isoforms, c-Src and KSR-1) along with core components of the ERK cascade (such as MEK and ERK) (Farshori et al., 2003; Dobkin-Bekman et al., 2009). Experiments in HEK293 cells have also shown that GnRH signals to ERK via a complex including focal adhesion kinase and c-Src in the cytosol (Davidson et al., 2004a,b). Despite their importance, the regulators that control ERK activity and localisation in the cell nucleus (as opposed to the cytosol) in response to GnRH signalling remain relatively poorly studied.

**Fig. 3:**
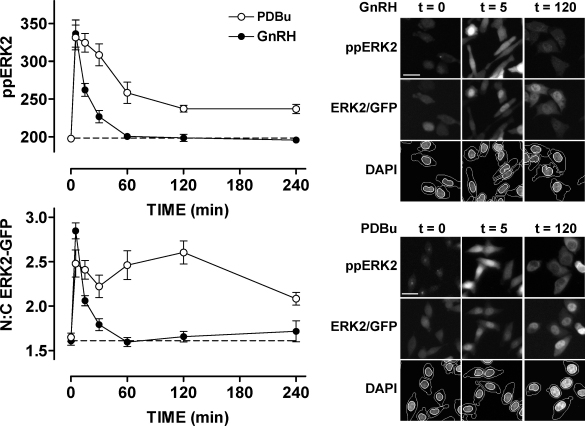
Spatiotemporal characteristics of GnRH and PDBu-stimulated ERK regulation revealed using an ERK knock-down and add-back model. Cells were transfected in 96-well plates with ERK1/2 siRNAs and transduced with Ad ERK2-GFP and Ad mGnRHR prior to stimulation with 10^−6^ M GnRH or PDBu for the times indicated. They were then fixed and stained before image acquisition and analysis for the calculation of whole-cell ppERK2 intensity (upper left panel) and the N:C ERK2-GFP ratio (lower left panel). Representative regions of cell images are also shown for DAPI, ERK2-GFP and ppERK2 in cells stimulated with 10^−6^ M GnRH or PDBu as indicated (right panels). Note that in spite of comparable initial responses appreciable levels of ppERK2 and nuclear retention of ERK2-GFP are only seen at 120 min in the PDBu stimulated cells (scale bars: 30 μm).

Adapted from Armstrong et al. (2009c).

Many ERK activating stimuli increase expression of nuclear-inducible dual-specificity phosphatases (DUSPs) and GnRH has been shown to increase expression of DUSP1 and 4 in gonadotrophs, but their full effect on ERK signalling is unclear (Zhang et al., 2001a,b; Davidson et al., 2004b). The potential complexity of this system is illustrated by the fact that ERK is activated by a single kinase (MEK), but can be inactivated by at least 13 phosphatases. With this in mind we have developed HCM methods for exploring how DUSPs may shape GnRH-mediated ERK signalling (Caunt et al., 2008a,b; Armstrong et al., 2009c). This involves staining cells in 96-well plates after treatment with antibodies to both ERK and dual-phosphorylated (pp) ERK and a DAPI stain for DNA. Automated image acquisition and analysis then provides a high throughput method of comparing ERK and ppERK compartmentalisation (Fig. 3). We have further developed a method for studying ERK function in which siRNAs (targeted to non-coding regions) are used to remove endogenous ERK1/2, and Ad are used to express either GFP-tagged wild-type ERK2, or a mutated allele of ERK2 to probe function. This allows live cell studies of ERK traffic or a counterstain for ppERK in the same cells can be included. The removal of endogenous ERK1/2 is important, firstly to allow staining for ppERK without interference from endogenous ERK1/2 and secondly because overexpression of ERK can swamp binding partners and mask normal localisation changes. Using numerical filters, individual cells expressing sub- or super-physiological levels of ERK2 can be excluded from analysis to prevent bias of data towards a highly under or over-expressing subpopulation of cells. We used these methods to assess how MKPs and other DUSP family members contribute to the stimulus specificity of ERK responses to GnRH, EGF or the PKC-activating phorbol ester, PDBu (phorbol 12, 13 dibutyrate). Using siRNA knock-down of DUSPs in a model HeLa cell line prior to stimulation with GnRH, EGF or PDBu for acute or sustained periods, we found that 12 of 16 phosphatases tested affected either ERK localisation, compartmentalisation or phosphorylation state (Caunt et al., 2008a). When each variable of stimulus, readout or timing was taken into consideration, there was almost no redundancy of effect of the individual DUSP siRNAs (Caunt et al., 2008a). Specifically, we found that the nuclear family of MKPs (comprising DUSP1, 2, 4 and 5) constitute negative regulators of ERK activity in the nucleus in response to PKC activation, while the JNK/p38 family MKPs (DUSP10 and 16) are positive regulators of ERK (Caunt et al., 2008a). These findings were corroborated by using the “knock-down, add-back” system to introduce a D319N mutated ERK2-GFP construct, which abrogates binding to docking (D)-domain containing proteins (including all MKPs), and mimicked the effects of nuclear MKP knock down (Caunt et al., 2008a,b). While the majority of GnRH signalling to ERK is mediated by PKC in this model, we found firstly that GnRH-mediated ERK signalling kinetics were distinct from those mediated by PDBu, and that they were unaffected by DUSP1 or 4 knock-down (Armstrong et al., 2009c). However, we did find that GnRH-induced ppERK signals were potentiated either by D319N mutation of ERK or inhibition of protein synthesis, which indicates that signal termination is (at least in part) mediated by high turnover, D-domain containing phosphatases (Armstrong et al., 2009c). We additionally found that DUSP3, 5, 9, 10 and 16 were able to influence GnRH-mediated ERK phosphorylation and/or localisation (Armstrong et al., 2009c). These data indicate the need for further study into how the DUSPs regulate ERK activity in response to GnRH. They also highlight the fact that multiple endpoint and condition experiments are crucial in defining DUSP function in such systems.

## 7 Decoding GnRH pulse frequency

As noted above, GnRH is secreted in brief pulses. Pulse frequency varies under different conditions (i.e. through the menstrual cycle) and GnRH effects on its target cells are frequency-dependent. This was illustrated in early studies where constant GnRH suppressed LH and FSH secretion, whereas restoration of GnRH pulses restored gonadotropin secretion (Belchetz et al., 1978). Similarly, expression of genes for rodent LHβ, FSHβ and the GnRHR are all increased more effectively at low or intermediate GnRH frequency (brief pulses at 30–120 min intervals) than at high frequency (pulses at 8–30 min intervals) or with sustained stimulation (Dalkin et al., 1989; Weiss et al., 1990; Shupnik, 1990; Haisenleder et al., 1991; Kaiser et al., 1993; Yasin et al., 1995; Bedecarrats and Kaiser, 2003; Ferris and Shupnik, 2006). Pulsatile agonists can be used to stimulate gonadotropin secretion, whereas sustained agonist treatment ultimately reduces gonadotropin secretion and this underlies agonist efficacy against steroid hormone-dependent cancers (Conn and Crowley, 1994; Schally, 1999). Given its physiological and pharmacological relevance, there is a great deal of interest in the mechanisms underlying GnRH pulse frequency decoding and unique features of these receptors (above) provide a valuable model for exploring 7TM receptor mediated frequency decoding without the complications of G-protein-independent signalling or receptor desensitisation. Two of the major signalling pathways activated by GnRHR mediate frequency decoding in other systems. Thus, ERK-dependent transcription is dependent upon stimulus frequency in some models (Cullen and Lockyer, 2002), and the fact that targeted knock-down of ERKs causes infertility confirms the importance of this pathway in mediating responses to physiological (pulsatile) stimulation (Bliss et al., 2009). Similarly, GnRHR-mediated activation of the Ca^2+^/calmodulin pathway can affect gonadotropin subunit gene expression (Haisenleder et al., 2003a,b; Burger et al., 2008) and calmodulins are well established as frequency-decoders in other systems (Hanson et al., 1994; De Koninck and Schulman, 1998; Craske et al., 1999; Mermelstein et al., 2001). More recently, the NFAT, has been implicated in transcriptional regulation by GnRH (Oosterom et al., 2005; Lim et al., 2007; Gardner and Pawson, 2009), specifically in repression of the βFSH subunit gene (Lim et al., 2007). NFATs are transcription factors activated by Ca^2+^/calmodulin-dependent activation of the protein phosphatase calcineurin (which dephosphorylates NFAT) and their possible role in mediation of GnRH action is of particular interest in light of the well established role of NFATs as frequency decoders in other systems (Li et al., 1998; Dolmetsch et al., 1998; Tomida et al., 2003; Berridge, 2006). There is also the potential for cross-talk with the non-canonical Wnt/Ca^2+^ pathway as GnRH mediates phospho-inhibition of GSK3β which phosphorylates NFATs and thereby opposes their transcriptional activity (Gardner et al., 2007).

The simplest frequency-dependent signalling scenario is one in which a train of brief stimuli elicits a series of corresponding responses in a process known as digital tracking (Berridge, 2008). However, down-stream responses are typically activated and inactivated more slowly than upstream signals so responses may not have returned to the pre-stimulation base-line before repeat stimulation. This can yield saw-tooth or cumulative responses (Krakauer et al., 2002; Ferris and Shupnik, 2006; Berridge, 2008) in a process known as integrative tracking. This can provide signal specificity and amplify signalling but cannot explain the bell-shaped frequency–response relationships often seen with pulsatile stimulation paradigms. These require positive or negative, feed-back or feed-forward loops (Krakauer et al., 2002). The lack of rapid homologous type I mammalian GnRHR desensitisation excludes one potential feedback mechanism but agonists do stimulate GnRHR internalisation and thereby reduce cell surface GnRHR number (Willars et al., 1999; Heding et al., 2000; Hislop et al., 2005; Caunt et al., 2006b; Finch et al., 2009). Sustained GnRH also down-regulates IP_3_ receptors (Willars et al., 2001; Wojcikiewicz et al., 2003) and increases regulator of G-protein signalling-2 (RGS2) expression, raising the possibility that this inhibits G_q/11_ signalling (Wurmbach et al., 2001; Karakoula et al., 2008). Similarly, GnRH increases expression of DUSPs that could generate feedback loops contributing to the frequency-decoding (Zhang et al., 2001a,b; Zhang and Roberson, 2006; Armstrong et al., 2009c). Alternatively, it has been proposed that frequency decoding at the LHβ promoter involves Egr-1 and a co-regulator (Nab-2). In this model, low GnRH frequency causes transient Egr-1 expression and consequent expression of Nab-2 which inhibits LHβ expression. Whereas at high frequency there is a more sustained increase in Egr-1 and this quenches Nab-2, increasing LHβ expression (Lawson et al., 2007). Similar interplay between c-fos and the co-regulator TGIF may also underlie preferential activation of the FSHβ promoter at low GnRH pulse frequency (Tsutsumi and Webster, 2009). Alternatively, a recent study revealed that GnRH-induced expression of ICER (inducible cAMP early repressor) antagonised the stimulatory effect of CREB (cAMP reponse element binding protein) to specifically inhibit FSHβ expression at high pulse frequency (Ciccone et al., 2009).

**Fig. 4:**
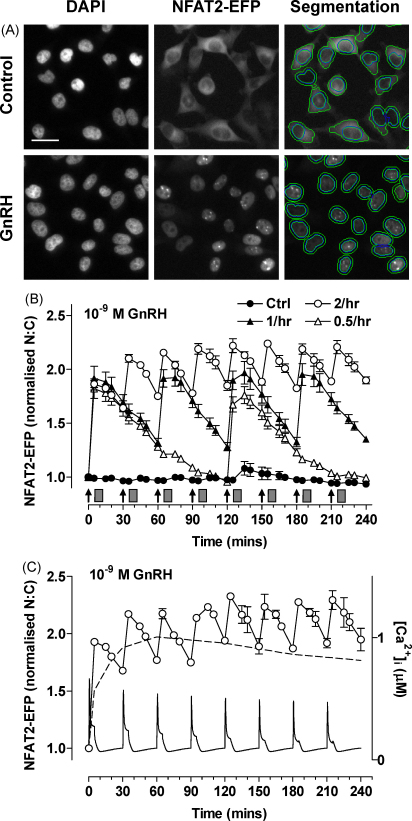
Live cell imaging with varied GnRH pulse frequency. Panel A: HeLa cells were transduced with Ad-mGnRHR and Ad-NFAT2-EFP and treated with 0 or 10^−7^ M GnRH for 20 min, washed with ice-cold PBS, fixed with 4% PFA, permeabilised and stained with DAPI. The panel shows representative images of cells acquired in the DAPI (blue) and EFP (green) image channels, with an example of the automated image segmentation used to define perimeters of nuclei and cells. Scale bar: 30 μm. Panel B: cells transduced with Ad-mGnRHR, Ad-NLS-BFP and Ad-NFAT2-EFP were treated with 10^−9^ M GnRH for 5 min at 30 min intervals, hourly intervals, or every 2 h, as indicated. All the wells were subject to half hourly washes (grey rectangles) 5 min after GnRH or control addition. Digital images were acquired from live cells and used to calculate the nuclear:cytoplasmic (N:C) ratio which was normalised to the control value obtained at time 0 in each well. Note that integrative tracking (i.e. the saw-tooth response seen when responses have not returned to control values before repeat stimulation) occurred at the highest pulse frequency. Panel C shows the response to 30 min pulses of 10^−9^ M GnRH along with the response seen in cells receiving constant stimulation with 10^−9^ M GnRH throughout the 4 h experiment (dotted line) and the underlying [Ca^2+^]_i_ estimated using an established mathematical model for GnRH signalling (Washington et al., 2004).

Adapted from Armstrong et al. (2009b).

A fundamental question raised by the data outlined above is whether or not feedback effects shaping cytoplasmic signals are actually relevant to GnRH frequency decoding. We have begun to address this using an NFAT2-EFP live cell imaging reporter (Armstrong et al., 2009b). We found that GnRH causes translocation of NFAT2-EFP from the cytoplasm to the nucleus and that this provides a robust readout for GnRHR-mediated activation of Ca^2+^/calmodulin/calcineurin/NFAT signalling. The effect was reversible but was slower in onset and offset than the underlying change in [Ca^2+^]_i_, and pulsatile GnRH caused dose- and frequency-dependent NFAT2-EFP translocation (Fig. 3). At low pulse frequency NFAT2-EFP translocation simply tracked GnRHR occupancy but integrative tracking occurred at high frequency (pulses every 30 min), illustrating how relative dynamics of upstream and downstream signals can increase efficiency of cellular response to pulsatile GnRH (Fig. 3). We also used a published mathematical model of GnRH signalling to predict responses during pulsatile stimulation. This predicted desensitisation of GnRHR-mediated effects on [Ca^2+^]_i_ and that such desensitisation would increase with dose, pulse frequency and receptor number (Armstrong et al., 2009b). However, no such desensitisation was seen (using the NFAT2-EFP reporter) in HeLa or LβT2 cells, possibly because pulsatile GnRH did not reduce cell surface GnRHR expression (Armstrong et al., 2009b). GnRHR activation also caused dose- and pulse frequency-dependent activation of αGSU-, LHβ- and FSHβ-luciferase reporters and each of these responses was prevented by cyclosporin A, indicating dependence upon the Ca^2+^/calmodulin/calcineurin pathway. Pulsatile GnRH also activated an NFAT-responsive luciferase reporter but its effect was directly related to cumulative pulse duration. This, together with the fact that we saw no desensitisation of the NFAT2-EFP translocation responses argues that although NFATs may mediate GnRH action, they are not genuine decoders of GnRH pulse frequency. We have also used a similar approach (ERK2-GFP imaging) to test for possible feedback regulation of GnRH-mediated ERK responses during pulsatile stimulation (Armstrong et al., 2009a). As expected, GnRH caused translocation of ERK2-GFP from the cytoplasm to the nucleus providing a robust, live-cell readout for GnRHR-mediated Raf/MEK/ERK activation. The effect was reversible and pulsatile GnRH caused dose- and frequency-dependent ERK2-GFP translocation (Armstrong et al., 2009a). These responses were faster in onset and offset than the GnRHR-mediated NFAT2-EFP translocation responses and showed only digital tracking of GnRHR occupancy (Fig. 4). Importantly, we saw no evidence for desensitisation of GnRH effects on ERK2-GFP translocation under any condition tested (dose, frequency and receptor number varied). Moreover, GnRH caused a frequency-dependent activation of an Egr1-responsive luciferase reporter (used as readout for ERK activation) but the response was directly related to cumulative pulse duration (Armstrong et al., 2009a).

The data outlined above suggest that frequency decoding cannot be attributed to feedback effects shaping Ca^2+^/calmodulin/calcineurin/NFAT or Raf/MEK/ERK signalling. An obvious caveat is that much of the live cell imaging was performed in HeLa cells and with relatively short periods of stimulation (maximally 8 h). However, it is important to recognise that genuine GnRH frequency decoding does occur under these conditions (as evidenced by bell-shaped frequency–response relationships for GnRH effects on LHβ-luc and FSHβ-luc reporters in this model) and that where examined, the data obtained with the imaging reporters was very similar in HeLa and LβT2 (gonadotroph lineage) cells (Armstrong et al., 2009b). Since we have found no evidence for genuine frequency decoding in these pathways, our data are consistent with two alternative possibilities, (a) that frequency decoding occurs within other upstream signalling pathways or (b) that frequency decoding occurs downstream of these pathways. The latter possibility is the cornerstone of models where differential regulation of FSHβ and LHβ expression is attributed to the interplay of transcription factors and co-regulatory proteins (Lawson et al., 2007; Ciccone et al., 2009; Tsutsumi and Webster, 2009).

## 8 Conclusions and future directions

GnRHR are structurally and functionally unique. Notably, they have undergone a relatively recent period of accelerated molecular evolution in which the advent of mammals has coincided with the loss of C-terminal tails and associated functions including rapid desensitisation, agonist-induced phosphorylation and arrestin-mediated signalling. The discovery that type I mammalian GnRHR do not desensitise underlines the importance of cell surface GnRHR number in determining responsiveness to GnRH, just as the discovery that most hGnRHR are intracellular, underlines the importance of compartmentalisation in determining cell surface GnRHR number. However, relatively little is known about the physiological relevance and molecular determinants of GnRHR trafficking to or from the cell surface. For example, we do not know the proportion of GnRHR at the cell surface in human gonadotrophs or whether this varies through the menstrual cycle, through puberty or at other developmental stages. Similarly, we know that a membrane permeant antagonist can increase the proportion of hGnRHR at the cell surface, and that addition of a XGnRHR C-tail to the hGnRHR has a similar effect; yet the sorting proteins detecting these differences in conformation or primary structure are largely unknown. We also know that agonists cause internalisation and down-regulation of cell surface type I mammalian GnRHR but mechanisms have so far been defined largely in negative terms (independence from receptor phosphorylation, arrestin binding and dynamin activity). The means by which agonists target these receptors for internalisation remain unknown. The high throughput provided by automated cell imaging will undoubtedly facilitate work on these issues and has already led to the surprising observation that cetrorelix, a compound that acts as a competitive GnRHR antagonist in many functional assays, is actually an inverse antagonist for GnRHR internalisation. Most importantly, this work demonstrates the occurrence of ligand biased efficacy with hGnRHR and in gonadotroph lineage cells, supporting the notion that it may prove to be physiologically and/or therapeutically relevant. Finally, we have found that automated imaging of fluorescent protein reporters provides a powerful means of interrogating GnRHR signalling to the Raf/MEK/ERK and Ca^2+^/calmodulin/calcineurin/NFAT cascades. This has facilitated live cell imaging of signalling during pulsatile GnRH stimulation, an approach that we consider essential in addressing the fundamental and long-standing question of how cells decode GnRH pulse frequency. To date our work has revealed how integrative tracking can increase the efficiency of target cell responsiveness to GnRH but also that these cascades appear not to decode frequency (at least in HeLa and LβT2 cells). Again, we are optimistic that HCM approaches will prove valuable in addressing alternatives, including the possibility that frequency decoding is an inherent feature of alternative upstream signals or an emergent feature of the network of signals passing from the cytoplasm to the transcriptome.

## Conflict of interest

None.

## Figures and Tables

**Table 1:** Ligand effects on GnRHR signalling and trafficking.

	High affinity binding	Signalling to Gq/11	Anterograde trafficking	Retrograde trafficking	Effect on PCSE
Unliganded GnRHR	N/A	None	Slow	Slow	N/A
+GnRH or Buserelin	Yes	↑↑	–	↑↑	↓↓
+Cetrorelix	Yes	–	–	↓	↑
+IN3	Yes	–	↑↑	–	↑↑

The table summarises data obtained by HCM in HeLa, MCF7 and LβT2 cell models as described in the text. In terms of cell surface receptor signalling via Gq/11, GnRH and buserelin are pure agonists whereas cetrorelix and IN3 are pure antagonists. The non-peptide antagonist IN3 increases anterograde trafficking (of hGnRH, h.XGnRHR and mGnRHR) to the plasma membrane whereas the membrane impermeant peptides (GnRH, buserelin and cetrorelix) have little or no effect on this parameter. In contrast, GnRH and buserelin increase retrograde trafficking (internalisation) of GnRHR whereas cetrorelix can slow it and IN3 has little or no effect. These functional characteristics of cell surface GnRHR cannot be explained with a conventional model assuming just 2 GnRHR conformations (active and inactive). The three distinct functional profiles (unliganded versus GnRH/buserelin occupied versus cetrorelix occupied) implies the existence of at least 3 conformations of cell surface GnRHR. These data support the notion that GnRHR show ligand biased efficacy and that the phenomenon is relevant to ligands used therapeutically. Note also that IN3 has the potential to increase cell surface GnRHR number but also to increase the number of GnRHR at the cell surface and its overall effect on GnRH signalling reflects the balance of these two effects. We have not yet observed any functional correlate of the more modest increase in cell surface GnRHR expression caused by the peptide antagonist cetrorelix. N/A = non-applicable. Arrows indicate whether the parameter is increased or decreased and “–” indicates that the measure is unchanged (as compared to the unliganded receptor).
